# Adaptive restructuring of the microbiota promotes overwintering survival of the tick *Dermacentor silvarum*

**DOI:** 10.1186/s13071-026-07457-3

**Published:** 2026-06-02

**Authors:** Tingwei Pei, Chuks F. Nwanade, Zhen Wang, Zifeng Liu, Yingying Dai, Xiaonan Zhang, Zhijun Yu

**Affiliations:** 1 Hebei Key Laboratory of Animal Physiology, Biochemistry and Molecular Biology, Hebei Collaborative Innovation Center for Eco-Environment, Hebei Research Center of the Basic Discipline of Cell Biology, Ministry of Education Key Laboratory of Molecular and Cellular Biology, College of Life Sciences, Hebei Normal University, Shijiazhuang, 050024 China; 2 Guangdong Key Laboratory of Animal Conservation and Resource Utilization, Guangdong Public Laboratory of Wild Animal Conservation and Utilization, Institute of Zoology, Guangdong Academy of Sciences, Guangzhou, China

**Keywords:** *Dermacentor silvarum*, Microbiota, Fungal community, Cold stress, Overwinter adaptation

## Abstract

**Background:**

*Dermacentor silvarum* is a medically important tick species in temperate regions of Asia that must survive prolonged cold exposure during winter. However, the impact of low-temperature stress on its microbial community and the potential functional implications for overwintering remain poorly understood.

**Methods:**

Adult ticks of *D. silvarum* were subjected to controlled low-temperature treatments (8, 4, 0, and −4 °C) for 7 days, with ticks maintained at 27 °C serving as controls. The bacterial 16S rRNA (V4 region) and fungal ITS1 regions were amplified and sequenced using the Illumina NovaSeq PE250 platform. Bioinformatics analyses were performed to assess microbial diversity, community composition, and predicted functional profiles.

**Results:**

Cold exposure significantly altered both bacterial and fungal community structures, increasing overall microbial diversity. Proteobacteria and Actinobacteriota dominated the bacterial assemblages, whereas the Ascomycota and Basidiomycota were the predominant fungal phyla. A marked enrichment of psychrotolerant and metabolically versatile genera, including *Pseudomonas*, *Sphingomonas*, and *Trichosporon*, alongside a decline in the nutrient-provisioning symbiont *Coxiella* were observed in cold-treated ticks. Functional prediction suggested that the enriched taxa are potentially involved in antioxidative defense, cryoprotectant biosynthesis, membrane stabilization, and detoxification processes.

**Conclusions:**

The results demonstrate that low temperature drives a comprehensive reorganization of the microbiota in *D. silvarum*. The increase in psychrotolerant and detoxifying microbes likely reflects an adaptive host–microbe interaction that enhances tolerance to cold, oxidative, and osmotic stress, thereby promoting overwinter survival of the ticks. These findings provide new insights into the ecological resilience of tick-associated microbiomes and the symbiotic mechanisms underlying vector adaptation to climatic challenges.

**Graphical Abstract:**

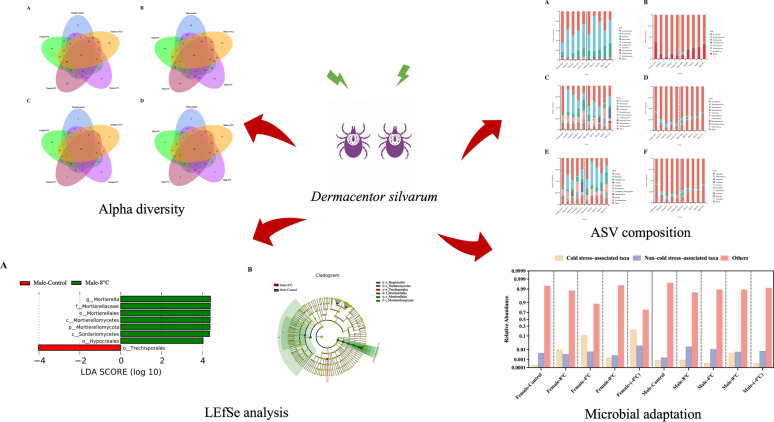

**Supplementary Information:**

The online version contains supplementary material available at 10.1186/s13071-026-07457-3.

## Background

Ticks are hematophagous arthropods that parasitize a wide range of vertebrate hosts, including humans, domestic animals, and wildlife [1]. As vectors of numerous zoonotic pathogens, ticks pose significant threats to public health and livestock production globally [2]. Among the approximately 960 tick species described worldwide, *Dermacentor silvarum* is a medically and veterinary important tick species widely distributed across temperate regions of northern China, Mongolia, and parts of Russia. It transmits several zoonotic pathogens, including *Rickettsia raoultii*, *Anaplasma phagocytophilum*, *Babesia venatorum*, and *Francisella tularensis*, which cause severe tick-borne diseases in humans and animals [3, 4].

Tick-associated microorganisms include both pathogenic and nonpathogenic taxa that engage in diverse interactions with their hosts, ranging from parasitism and commensalism to mutualism [5]. These microbial communities not only affect the vector competence of ticks, but also influence their physiology, development, reproduction, and ecological adaptability [6]. Increasing evidence suggests that microbial diversity in arthropods is shaped by multiple biotic and abiotic factors, such as host genetics, symbiont interactions, developmental stage, feeding status, and environmental conditions [7]. Among these, ambient temperature is a key ecological driver that modulates the diversity, stability, and functional capacity of the host microbiome [8].

Temperature profoundly shapes ecological communities and their constituent organisms, including arthropods. Elevated temperatures may either enhance or suppress insect immune defenses against microorganisms, thereby altering host susceptibility to antagonistic microorganisms, while both chronic and acute thermal stress can modulate immune function [9]. In many ectothermic organisms, including arthropods, temperature has been shown to profoundly impact microbial composition. For instance, transient thermal stress can rapidly alter the gut microbiota of *Drosophila subobscura*, which in turn affects host thermal tolerance [10]. In leafcutter ants *Atta texana*, expansion into colder northern climates was facilitated through the acquisition of cold-tolerant mutualistic fungi [11]. Similarly, tick-associated microbiota have demonstrated sensitivity to temperature fluctuations [12, 13]. For example, the microbial community of the blacklegged deer tick *Ixodes scapularis* exhibited significant compositional changes when maintained at different temperatures (4–37 °C) under laboratory conditions [12], and small-scale environmental variations were shown to shape the microbiota of the sheep tick *Ixodes ricinus* in the field [13].

Despite increasing interest in tick–microbiome interactions, knowledge about how overwintering temperatures affect the microbial diversity of ticks remains limited. In cold temperate regions, ticks must endure extended periods of low temperature during winter quiescence or diapause, and their associated microbiota may play important roles in host survival by enhancing cold tolerance, regulating metabolism, or modulating stress responses. However, the dynamics of these microbial communities under cold stress remain poorly understood.

In this study, the composition and diversity of bacterial communities in *D. silvarum* exposed to different low-temperature treatments was investigated using high-throughput 16S rRNA gene sequencing on the Illumina NovaSeq PE250 platform. By comparing the microbial profiles across varying cold exposure conditions, we aimed to elucidate how low temperatures shape tick-associated microbiota. Our findings provide novel insights into the ecological and functional roles of microbial communities during tick overwintering and may inform new strategies for the control of ticks and tick-borne diseases under changing climatic conditions.

## Methods

### Tick sampling and processing

Unfed adult *D. silvarum* ticks were sampled by flag-dragging vegetation in the Xiaowutai National Nature Reserve, Hebei Province, China. The ticks were brought back to the laboratory and reared on the ears of white rabbits (*Oryctolagus cuniculus*) (each rabbit was used for a single infestation). During the off-host period, the ticks were maintained in environmental chambers under controlled conditions (27 ± 2 °C, 80 ± 5% relative humidity, 16 h light: 8 h dark). Adult ticks that were 2 weeks post-molt from the second laboratory generation were used for subsequent assays. All experiments were approved by the Animal Ethics Committee of Hebei Normal University (protocol number: IACUC-223815).

For cold exposure assays, groups of 20 males or 20 females were randomly selected and transferred into 1.5 mL centrifuge tubes. Then, they were abruptly exposed and incubated at 8, 4, 0, or −4 °C for 7 days, which represent ecologically relevant low temperatures that ticks commonly encounter during winter in their natural habitats [14, 15]. Ticks maintained at 27 ± 2 °C served as the control group, and the relative humidity was controlled using saturated salt solutions. Each treatment was conducted in triplicate, with 60 ticks used per treatment. After exposure, ticks were surface-sterilized by three washes in 70% ethanol followed by one wash with sterile nuclease-free deionized water to eliminate environmental contaminants [16, 17]. The ticks from each replicate were then collected into 1.5 mL centrifuge tubes, flash-frozen in liquid nitrogen, and stored at −80 ℃ until further analysis.

### DNA extraction and 16S rRNA sequencing

After temperature treatments, ticks were surface-sterilized as described above and then ground using 0.2 g zirconia beads (0.5 mm, Maiang, China) in a bead mill. Genomic DNA (gDNA) was extracted using the DNeasy Blood & Tissue Kit (Qiagen, Germany) according to the manufacturer’s instructions. The bacterial 16S rRNA V4 region and the fungal internal transcribed spacer 1 (ITS1) region were amplified to profile microbial communities. DNA extracted from each sample was used as the template for polymerase chain reaction (PCR) on a ProFlex PCR Thermal Cycler (Thermo Fisher Scientific, USA). Specific barcoded primer sets were used to amplify bacterial (515F: 5′-GTGCCAGCMGCCGC GGTAA-3′; 806R: 5′-GGACTACHV GGGTWTCTAAT-3′) [18] and fungal (ITS1-1F-F: 5′-CTTGGTCATTTAGAGGA AGTAA-3′; ITS1-1F-R: 5′-GCTGCGTTCTTCATCG ATGC-3′) [19, 20] targets.

Each amplification reaction (30 μL) comprised 15 μL of Phusion® High-Fidelity PCR Master Mix with GC buffer (New England Biolabs, UK), 0.5 μM of each primer, approximately 10 ng of genomic DNA, and nuclease-free water. The thermal cycling protocol for the bacterial 16S region included an initial denaturation at 98 °C for 30 s, followed by 30 cycles of 98 °C for 15 s, 55 °C for 30 s, and 72 °C for 2 min, with a final extension at 72 °C for 5 min. For ITS1 amplification, cycling conditions were set at 98 °C for 1 min, followed by 30 cycles of 98 °C for 20 s, 53 °C for 30 s, and 72 °C for 1 min, ending with a 10 min extension at 72 °C.

PCR products were confirmed by electrophoresis on 2% agarose gels, and amplicons of the expected sizes (approximately 400–450 bp for 16S and 300–350 bp for ITS1) were excised and purified using the GeneJET™ Gel Extraction Kit (Thermo Fisher Scientific, USA). The purified fragments were quantified using a Qubit 3.0 fluorometer (Thermo Fisher Scientific). For each sample, 10–40 ng of DNA was used for library construction. Libraries were prepared using the NEBNext® Ultra™ II DNA Library Prep Kit (New England Biolabs, USA) following the manufacturer’s instructions. The constructed libraries were quantified using a Qubit® 2.0 Fluorometer (Thermo Fisher Scientific, USA) and quantitative (q)PCR. Paired-end sequencing (PE250) was conducted on an Illumina NovaSeq 6000 platform at Novogene Bioinformatics Technology Co., Ltd. (Tianjin, China).

The raw bacterial and fungal sequence data were submitted to the NCBI Sequence Read Archive (SRA) under BioProject accession numbers PRJNA1069662 and PRJNA1092565, respectively.

### Bioinformatics analysis

Paired-end reads were merged using FLASH (Version 1.2.11) [21], and raw tags were quality-filtered with fastp (Version 0.20.0) to obtain high-quality clean tags. Chimera detection was performed using Vsearch (Version 2.15.0) [22] against the SILVA (Version 138.1) (https://www.arbsilva.de/ for 16S/18S) [23] and UNITE (Version 8.2) (https://unite.ut.ee/ for ITS) [24] databases, and chimeric sequences were removed. The denoising of clean reads was conducted using the full amplicon workflow in QIIME2 (Version QIIME2-202,006) with either DADA2 [25], generating high-resolution amplicon sequence variants (ASVs). Low-abundance ASVs with fewer than five reads were excluded from downstream analysis.

Taxonomic assignment of ASVs was performed in QIIME2 using a naïve Bayes classifier trained on the SILVA database for bacterial sequences and the UNITE database for fungal sequences. Multiple sequence alignment was conducted in QIIME2 to infer phylogenetic relationships among ASVs. To account for uneven sequencing depth, the data were rarefied to the lowest sequencing depth across all samples prior to diversity analysis.

Alpha-diversity indices, including Shannon diversity [26], Simpson index [27], Chao1 richness [28], observed species, Good’s coverage [29], and Faith’s phylogenetic diversity [30], were calculated using the QIIME2 pipeline. The adonis and anosim functions in the QIIME2 software were used to explore the significance of the differences in community structure between groups. Rarefaction curves and species accumulation curves were generated to evaluate sequencing depth and species richness. Beta-diversity was assessed on the basis of Bray–Curtis dissimilarity as well as weighted and unweighted UniFrac distances, and nonmetric multidimensional scaling (NMDS) was used to visualize microbial community structure.

Venn diagrams were generated in R (Version 3.5.3) (http://www.R-project.org/) [31] to visualize the shared and unique ASVs among different treatment groups. Linear discriminant analysis effect size (LEfSe) was applied to identify differentially enriched taxa, using the Kruskal–Wallis test with a linear discriminant analysis (LDA) score threshold of > 4. Functional prediction of bacterial communities was performed using Phylogenetic Investigation of Communities by Reconstruction of Unobserved States 2 (PICRUSt2) on the basis of the Kyoto Encyclopedia of Genes and Genomes (KEGG) database. This approach infers the functional potential of microbial communities by predicting gene family abundances and mapping them to KEGG Orthology (KO) groups and metabolic pathways. Fungal guild structures were annotated using FUNGuild to classify fungal taxa into ecological guilds on the basis of taxonomic information and published literature, rather than predicting metabolic pathways.

## Results

### Illumina NovaSeq 16S rRNA sequencing

A total of 2,428,365 raw reads were obtained from all female and male tick samples sequenced for bacterial 16S rRNA gene amplicons. The effective sequences had an average length of 412 bp, with the number of high-quality reads per sample ranging from 51,283 to 76,730. For the fungal ITS1 region, sequencing of 30 samples yielded 2,433,159 raw reads, of which 2,261,873 high-quality reads were retained after stringent quality filtering. The average effective sequence length was 202 bp, and the number of quality-filtered reads per sample ranged from 67,605 to 85,490 (Supplementary Table 1). Species accumulation curves for both bacterial and fungal communities (Supplementary Fig.  1 A, B) approached a clear asymptote, indicating that the sampling was sufficient to capture the majority of taxa present. Both bacterial and fungal rarefaction curves reached clear plateaus, indicating that sequencing depth and coverage were sufficient to capture the major microbial diversity present in all samples (Supplementary Fig.  2 A, B). Collectively, 3370 ASVs were detected across the 30 fungal samples (Supplementary Table 2).

A total of 5030 amplicon sequence variants (ASVs) were identified across all bacterial samples (Supplementary Table 3). Among the female samples, the highest numbers of unique ASVs were recorded in female ticks in the control group (662 ASVs) and those treated at 0 °C (363 ASVs) (Supplementary Fig.  3 A). In the male samples, ticks treated at 4 °C (685 ASVs) and at 0 °C (637 ASVs) exhibited the greatest numbers of unique ASVs (Supplementary Fig. 3B). The numbers of ASVs shared among the female and male groups were 55 and 61, respectively (Supplementary Fig.  3 A, B). Within the female samples, female ticks in the control group (456 ASVs) and female ticks treated at 8 °C (288 ASVs) contained the largest numbers of unique ASVs (Supplementary Fig.  3 C). In the male samples, ticks treated at 4 °C (402 ASVs) and ticks treated at −4 °C (351 ASVs) harbored the most unique ASVs (Supplementary Fig. 3D). The numbers of ASVs shared among female and male groups were 115 and 121, respectively (Supplementary Fig.  3 C, D).

**Fig 1.**
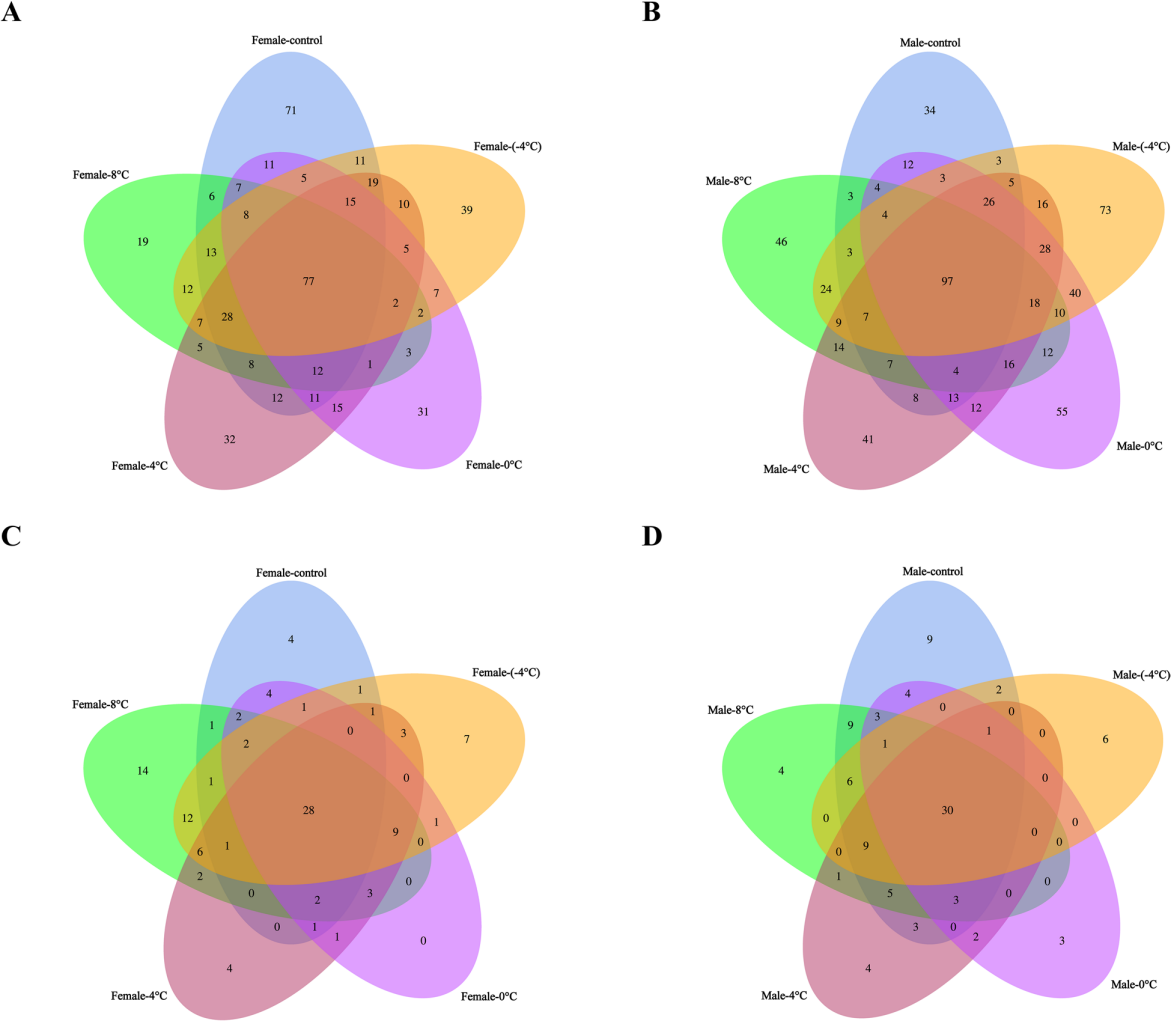
Venn diagrams of the microbiota in *Dermacentor silvarum*, showing the number of shared and unique taxa at the genus level among samples subjected to different low-temperature treatments and control groups. **A** Bacterial genera; **B** fungal genera

Venn diagram analysis at the genus level revealed similar distribution patterns among treatments (Fig. 1). Among the female samples, the control group (71 genera) and ticks exposed to −4 °C (39 genera) exhibited the highest numbers of unique genera (Fig. 1A). In the male samples, the largest numbers of unique genera were detected in ticks treated at −4 °C (73 genera) and 0 °C (55 genera) (Fig. 1B). A total of 77 and 97 genera were shared among the female and male groups, respectively (Fig. 1A, B).

For the fungal communities, ticks treated at 8 °C showed the highest number of unique genera in the female samples (14 genera), whereas the control group contained the highest number in the male samples (5 genera) (Fig. 1C, D). The numbers of shared genera were 28 and 30 in the female and male groups, respectively.

### Richness and diversity of bacterial and fungal species

**Fig 2.**
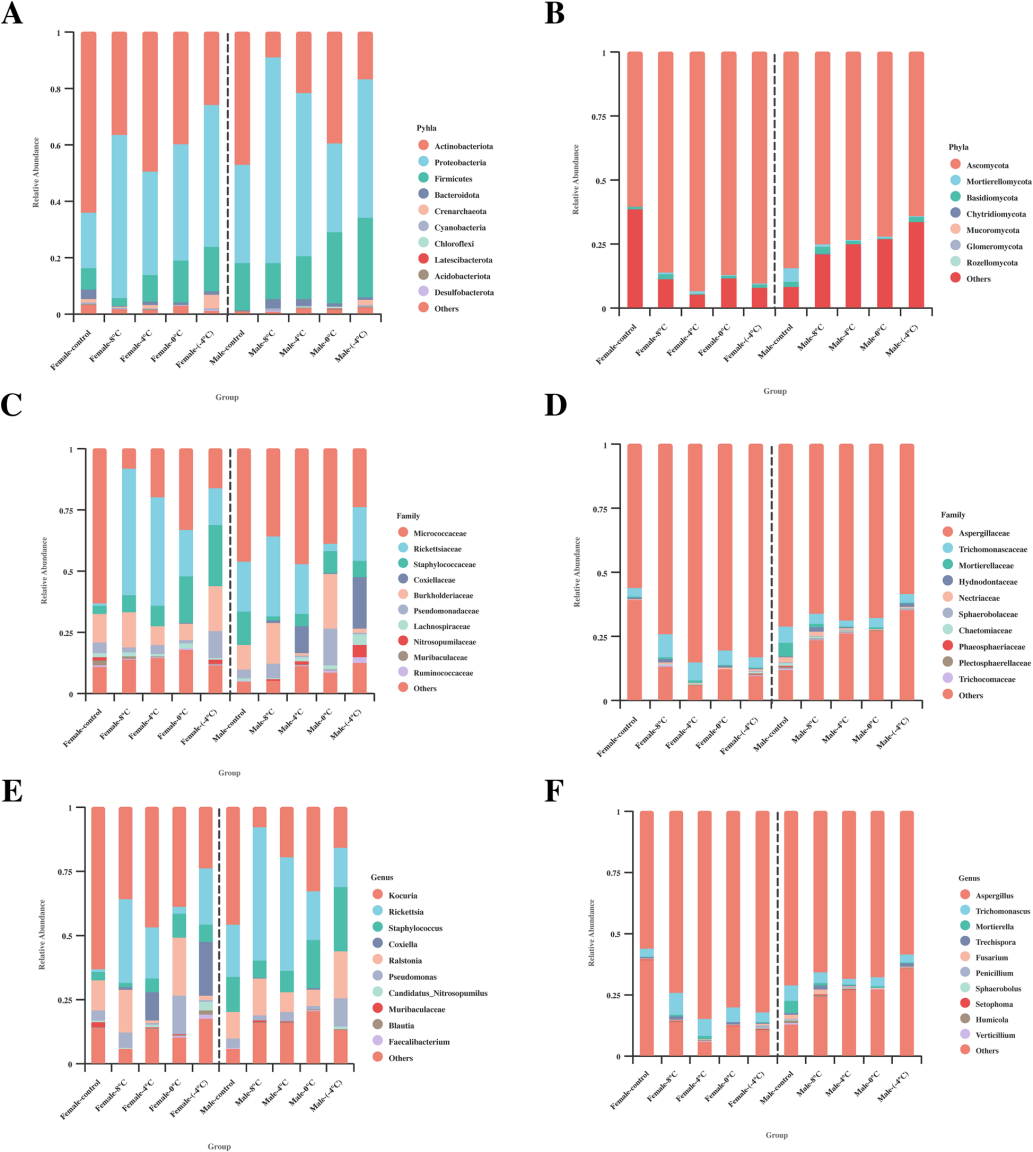
Relative abundance of the top ten most abundant taxa in *Dermacentor silvarum* at multiple taxonomic levels across low-temperature treatments and control groups. Shown are bacterial phyla (**A**), fungal phyla (**B**), bacterial families (**C**), bacterial genera (**D**), fungal families (**E**), and fungal genera (**F**)

At the phylum level, a total of 38 bacterial phyla were identified across all samples (Supplementary Table 4). Among these, Proteobacteria, Actinobacteria, and Firmicutes were the predominant phyla, together contributing more than 80% of the total bacterial community composition. Proteobacteria exhibited the highest relative abundance in most groups, particularly in female ticks treated at 8 °C (73.70%), and male ticks treated at 8 °C (76.60%) and at 4 °C (44.82%), whereas Actinobacteriota showed particularly high relative abundance in the control groups, accounting for 41.37% in female ticks and 76.65% in male ticks. Firmicutes was consistently the third most abundant phylum, showing 17.63% in female ticks in the control group, 31.74% in female ticks treated at 4 °C, and lower abundances in female ticks treated at 8 °C (4.30%), male ticks in the control group (0.21%), and male ticks treated at 4 °C (4.80%) and 8 °C (0.88%). Minor phyla, such as Bacteroidota, Acidobacteriota, Cyanobacteria, and Desulfobacterota, were detected at consistently low abundances (generally < 0.3%) across all groups (Fig. 2A). In the fungal community, eight phyla were identified across all samples (Supplementary Table 5). Ascomycota and Basidiomycota were the dominant phyla across all treatments. Ascomycota showed the highest relative abundance, ranging from 45.22% in female ticks treated at 8 °C to 76.42% in male ticks treated at 4 °C, whereas Basidiomycota represented the second most abundant phylum, with proportions varying from 21.89% in male ticks treated at 4 °C to 53.88% in female ticks treated at 8 °C. Other fungal phyla, including Mortierellomycota, Rozellomycota, Olpidiomycota, and unclassified fungi, were present at very low abundances (< 3%) in all samples (Fig. 2B).

A total of 375 bacterial families were identified across all samples (Supplementary Table 6). Among the top families, Coxiellaceae, Pseudomonadaceae, Vibrionaceae, Moraxellaceae, Enterobacteriaceae, Burkholderiaceae, Pseudoalteromonadaceae, and Comamonadaceae (Proteobacteria) were predominant, whereas only Lachnospiraceae (Firmicutes) and Muribaculaceae (Bacteroidota) appeared in the top ten families (Fig. 2C). At the genus level, 734 genera were detected (Supplementary Table 7). *Coxiella* was the dominant genus in the control groups (female ticks and male ticks in the control group), with consistently high relative abundances (86.91–97.85%). After treatment, marked shifts occurred, particularly in both female and male ticks treated at 8 °C, where *Pseudomonas* became dominant in several samples (37.17% in female ticks treated at 8 °C and 34.11% in male ticks treated at 8 °C), supplanting *Coxiella* as the dominant genus. Several Proteobacteria-associated genera, including *Vibrio*, *Acinetobacter*, *Ralstonia*, and *Pseudoalteromonas*, also increased following treatment. Low-abundance taxa from other phyla, such as Streptococcus and Lactobacillus (Firmicutes), Muribaculaceae (Bacteroidota), and Kocuria (Actinobacteria), were also detected across groups, exhibiting low but stable abundances (Fig. 2D). At the species level, *Coxiella* like endosymbiont was found to dominate the bacterial community across all sample groups (Supplementary Fig.  4 A, Supplementary Table 8).

For the fungal community, a total of 247 fungal families, 219 genera, and 283 species were identified across all samples (Supplementary Table 9–11). Among the top ten families, Aspergillaceae, Trichomonascaceae, Mortierellaceae, Hydnodontaceae, Nectriaceae, Sphaerobolaceae, Chaetomiaceae, Phaeosphaeriaceae, Plectosphaerellaceae, and Trichocomaceae were the most abundant, with the majority belonging to the phylum Ascomycota, whereas Hydnodontaceae and Sphaerobolaceae represented major components of Basidiomycota (Fig. 2E). Aspergillaceae showed the highest overall abundance across samples, followed by Trichomonascaceae and Mortierellaceae, both of which were consistently represented in nearly all treatment groups. Members of Hydnodontaceae and Nectriaceae also contributed substantially to the fungal community structure, particularly under low-temperature treatments.

At the genus level, *Aspergillus*, *Trichomonascus*, *Mortierella*, *Trechispora*, and *Fusarium* were among the most dominant taxa across samples (Supplementary Table 10). *Aspergillus* exhibited overwhelming dominance, reaching a relative abundance of 89.93% in female ticks treated at −4 °C. *Trichomonascus* also showed notable enrichment, particularly in male ticks treated at 8 °C, where it accounted for 16.20% of the fungal community. *Mortierella* was most abundant in male ticks in the control group (7.74%), whereas *Trechispora* reached its highest proportion in male ticks treated at 8 °C (4.78%). *Fusarium* displayed comparatively lower but consistent representation across treatments, with the maximum abundance observed in male ticks in the control group (2.88%) (Fig. 2F). Among the fungal species, *Aspergillus caesiellus*, *Aspergillus protuberus*, *Aspergillus penicillioides*, and *Trichomonascus ciferrii* were among the most abundant fungal species across the sample groups (Supplementary Fig. 4B).

**Fig 3.**
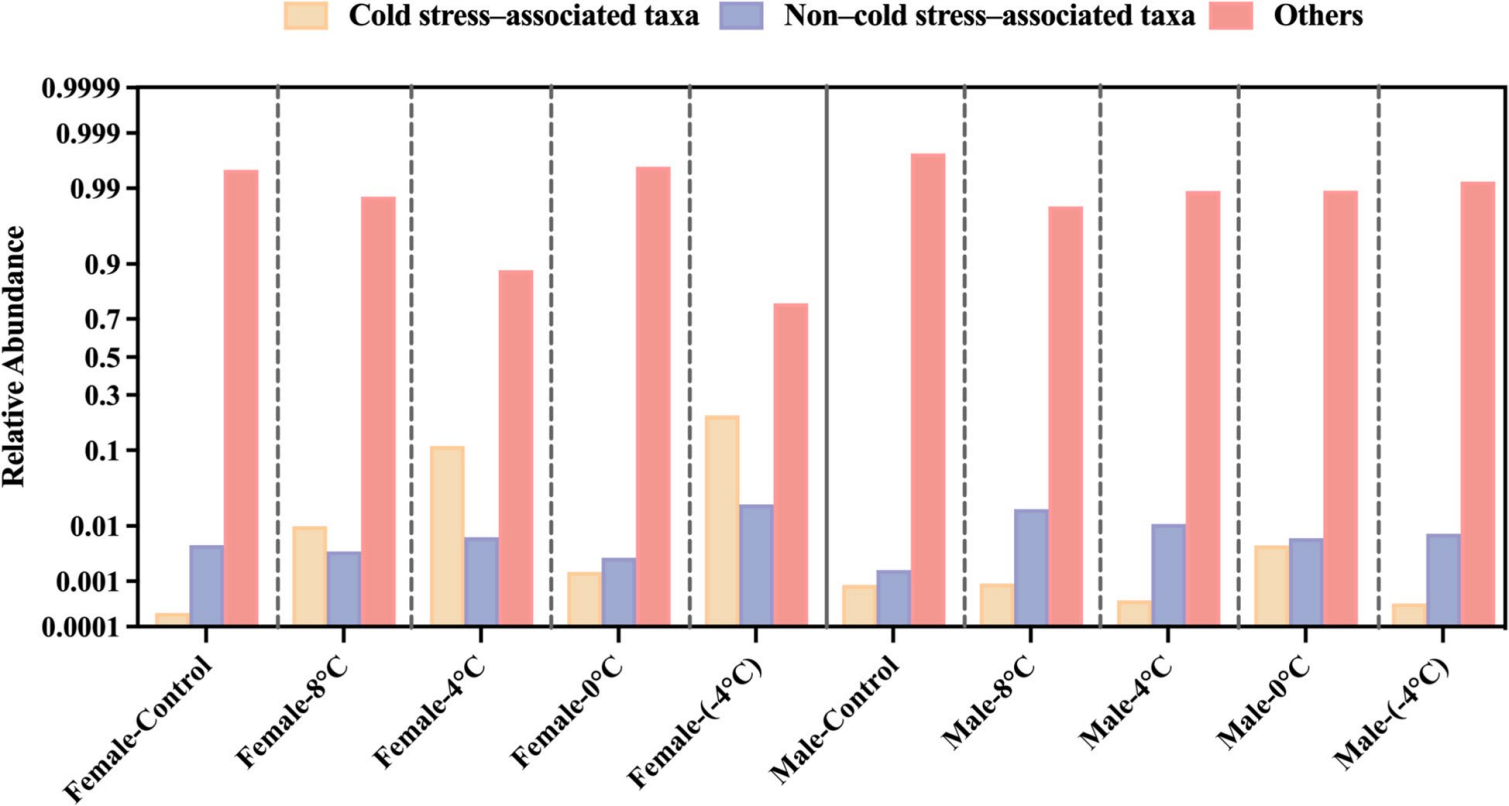
Changes in species-level bacterial community composition across low-temperature treatments (27 °C, 8 °C, 4 °C, 0 °C, and −4 °C). The top ten most abundant bacterial species were categorized into three functional groups: cold stress–associated taxa, represented by *Coxiella*-like endosymbionts (CLE); non-stress-related taxa; and “others” (representing the remaining species among the top ten in relative abundance)

As shown by the Shannon diversity index (Table 1), the bacterial diversity in *D. silvarum* ticks exposed to low temperature varied among the different treatment groups. The highest bacterial diversity was observed in samples of female ticks in the control group (Shannon = 5.263) and male ticks treated at 0 °C (Shannon = 4.875), whereas the lowest was recorded in female ticks in the control group (Shannon = 0.450) and female ticks treated at 8 °C (Shannon = 1.003). Similarly, the Simpson index values were higher in female ticks in the control group (0.857) and male ticks treated at 0 °C (0.841), indicating greater bacterial evenness in these samples compared with others. In contrast, dominance values were notably higher in female ticks in the control group (0.915) and female ticks treated at 8 °C (0.802), suggesting that the bacterial community in these samples was primarily driven by a few dominant taxa (Supplementary Table 12). For clarity of presentation, the analysis focused on the top ten bacterial species, which collectively represented more than 98% of the total microbial community across all samples, thus capturing the dominant ecological signals. These taxa were categorized into three functional groups: cold stress-associated taxa, represented by *Coxiella*-like endosymbionts (CLE); non-stress-related taxa; and a residual category termed “others.” Across all low-temperature treatments (8 °C, 4 °C, 0 °C, and −4 °C), the bacterial community was overwhelmingly dominated by CLE (Fig. 3). In female ticks, the relative abundance of CLE exhibited a general upward trend as temperatures decreased, despite a minor fluctuation at 0 °C, and reached its peak at −4 °C, indicating a broadly temperature-dependent response. In contrast, although male samples showed a slight increase in CLE abundance at 0 °C, no significant or consistent temperature-associated changes were detected across the full range of treatments. Meanwhile, the relative abundances of non-stress-related taxa and the “others” group showed only minor fluctuations and lacked a consistent trend across treatments, suggesting that these components of the microbiota remained comparatively stable under cold stress conditions.

**Table 1:** Alpha diversity parameters of the bacterial community in *Dermacentor silvarum* ticks exposed to low temperatures

Sample name		Chao1	Dominance	Goods coverage	Observed otus	Pielou-e	Shannon	Simpson
Female	Control	400.76 ± 351.58	0.6 ± 0.41	1.0 ± 0.0	396.67 ± 350.81	0.26 ± 0.25	2.4 ± 2.54	0.4 ± 0.41
	8 °C	201.75 ± 42.46	0.57 ± 0.23	1.0 ± 0.0	195.33 ± 40.46	0.22 ± 0.08	1.64 ± 0.62	0.43 ± 0.23
	4 °C	286.82 ± 38.45	0.31 ± 0.06	1.0 ± 0.0	284.0 ± 35.76	0.36 ± 0.04	2.95 ± 0.35	0.69 ± 0.06
	0 °C	264.0 ± 113.21	0.38 ± 0.18	1.0 ± 0.0	260.0 ± 113.86	0.33 ± 0.12	2.64 ± 1.18	0.62 ± 0.18
	−4 °C	316.88 ± 156.68	0.32 ± 0.11	1.0 ± 0.0	312.67 ± 152.36	0.41 ± 0.1	3.38 ± 1.1	0.68 ± 0.11
Male	Control	266.92 ± 136.21	0.35 ± 0.08	1.0 ± 0.0	260.0 ± 140.62	0.32 ± 0.07	2.52 ± 0.59	0.65 ± 0.08
	8 °C	313.89 ± 243.95	0.35 ± 0.16	1.0 ± 0.0	305.33 ± 244.06	0.36 ± 0.12	2.94 ± 1.41	0.65 ± 0.16
	4 °C	462.1 ± 210.29	0.37 ± 0.17	1.0 ± 0.0	458.0 ± 213.53	0.35 ± 0.12	3.13 ± 1.26	0.63 ± 0.17
	0 °C	379.44 ± 228.9	0.29 ± 0.11	1.0 ± 0.0	374.0 ± 225.48	0.39 ± 0.13	3.24 ± 1.42	0.71 ± 0.11
	−4 °C	365.73 ± 89.31	0.24 ± 0.05	1.0 ± 0.0	364.0 ± 89.27	0.39 ± 0.06	3.31 ± 0.59	0.76 ± 0.05

The fungal community also showed marked variations in alpha diversity among different samples (Table 2). The highest fungal diversity was observed in female ticks in the control group (Shannon = 4.742) and male ticks treated at 8 °C (Shannon = 4.611) and −4 °C (Shannon = 4.611), whereas the lowest was found in female ticks treated at 8 °C (Shannon = 1.844) and 4 °C (Shannon = 1.913). A similar pattern was observed in the Simpson index, with the highest values in female ticks in the control group (0.888) and in male ticks treated at 8 °C (0.891). The Chao1 richness estimator indicated that male ticks treated at −4 °C (Chao1 = 438.516) and 4 °C (Chao1 = 486.246) had relatively higher species richness compared with other samples (Supplementary Table 7). To further characterize the effects of temperature stress on microbial community composition, hierarchical clustering heatmaps were constructed on the basis of species-level annotation and normalized abundance data of the top 35 most abundant bacterial and fungal taxa across all samples. Both bacterial and fungal communities exhibited pronounced restructuring under temperature treatments (Supplementary Fig.  5 A, B). Clear separation was observed between treated groups and their corresponding controls in both female and male ticks, indicating consistent shifts in microbial composition in response to thermal stress. In particular, temperature exposure led to distinct clustering patterns that differentiated samples according to both treatment and sex, highlighting the combined environmental influences.

**Table 2:** The diversity parameters of the fungal community in *Dermacentor silvarum* ticks exposed to low temperatures

Sample name		Chao1	Dominance	Goods coverage	Observed otus	Pielou-e	Shannon	Simpson
Female	Control	444.69 ± 18.41	0.28 ± 0.15	1.00 ± 0.003	426.33 ± 12.86	0.43 ± 0.10	3.76 ± 0.86	0.72 ± 0.15
	8 °C	389.92 ± 67.06	0.29 ± 0.06	1.00 ± 0.003	367.00 ± 61.98	0.37 ± 0.02	3.10 ± 0.24	0.71 ± 0.06
	4 °C	353.72 ± 46.29	0.51 ± 0.04	1.00 ± 0.003	237.33 ± 43.43	0.25 ± 0.02	2.01 ± 0.22	0.49 ± 0.04
	0 °C	345.49 ± 73.13	0.43 ± 0.13	1.00 ± 0.003	320.00 ± 66.00	0.31 ± 0.08	2.57 ± 0.71	0.57 ± 0.13
	−4 °C	334.42 ± 108.41	0.36 ± 0.02	1.00 ± 0.003	307.33 ± 107.82	0.32 ± 0.04	2.61 ± 0.47	0.64 ± 0.02
Male	Control	322.72 ± 14.90	0.39 ± 0.05	1.00 ± 0.003	304.33 ± 20.60	0.36 ± 0.02	2.95 ± 0.22	0.61 ± 0.05
	8 °C	436.21 ± 50.39	0.33 ± 0.24	1.00 ± 0.003	317.00 ± 41.58	0.41 ± 0.14	3.39 ± 1.20	0.67 ± 0.24
	4 °C	428.96 ± 106.97	0.34 ± 0.21	1.00 ± 0.003	413.33 ± 105.08	0.40 ± 0.12	3.48 ± 1.16	0.66 ± 0.21
	0 °C	401.55 ± 40.25	0.33 ± 0.04	1.00 ± 0.003	375.67 ± 41.06	0.38 ± 0.02	3.23 ± 0.24	0.67 ± 0.04
	−4 °C	407.03 ± 89.96	0.18 ± 0.04	1.00 ± 0.003	394.67 ± 94.48	0.48 ± 0.06	4.10 ± 0.64	0.82 ± 0.04

### Variation in microbial abundance

The differences in abundance of the top ten bacterial and fungal genera between different temperature-treated tick groups were analyzed using Student’s *t*-tests (Supplementary Fig. 6A–I). Overall, temperature displayed a clear influence on the microbial community structure of ticks, with more pronounced changes observed under lower-temperature treatments.

For the bacterial community, significant differences were detected among several dominant genera. Across the temperature gradient, bacterial community changes were relatively limited at moderate temperatures (8 °C and 4 °C), whereas exposure to lower temperatures (0 °C) resulted in more pronounced shifts in specific genera. When comparing female ticks in the control group with those treated at 8 °C, Bifidobacterium (*P* = 0.023) exhibited marked changes in abundance (Supplementary Fig.  6 A). Between male ticks in the control group and those treated at 4 °C, the relative abundance of Sphingomonas (*P* = 0.012), Pseudarthrobacter (*P* = 0.049), and Shuttleworthia (*P* = 0.010) differed significantly (Supplementary Fig. 6B). Similarly, a comparison of male ticks in the control group with those treated at 0 °C revealed significant differences in Shuttleworthia (*P* = 0.005) and Succinivibrio (*P* = 0.017) (Supplementary Fig.  6 C).

For the fungal community, remarkable variations in relative abundance were also observed between treatment and control groups. Compared with the bacterial community, fungal taxa showed broader and more pronounced responses to temperature changes, particularly under low-temperature conditions. Among female ticks, relative to the control group, those treated at 4 °C showed significant differences in the abundance of Trichomonascus (*P* = 0.042) and Verticillium (*P* < 0.001) (Supplementary Fig. 6D). Females treated at −4 °C exhibited a significant change in Hamigera (*P* = 0.022) (Supplementary Fig. 6E), while those treated at 8 °C displayed a significant difference in Penicillium (*P* = 0.002) (Supplementary Fig.  6 F). In male ticks, comparison between the control group and those treated at 0 °C revealed significant differences in multiple genera, including Sphaerobolus (*P* = 0.033), Humicola (*P* = 0.019), Verticillium (*P* = 0.029), Talaromyces (*P* = 0.009), Trichoderma (*P* = 0.023), Sistotrema (*P* = 0.012), Saitozyma (*P* = 0.004), Clonostachys (*P* = 0.027), Scedosporium (*P* = 0.028), and Schizothecium (*P* = 0.044) (Supplementary Fig. 6G). Additionally, males treated at −4 °C showed significant changes in the relative abundance of Sphaerobolus (*P* = 0.033), Humicola (*P* = 0.018), Verticillium (*P* = 0.028), Trichoderma (*P* = 0.015), Saitozyma (*P* = 0.012), Clonostachys (*P* = 0.050), and Schizothecium (*P* = 0.038). Between the control group and males treated at 8 °C, significant differences were recorded for Verticillium (*P* = 0.033) and Sistotrema (*P* = 0.048) (Supplementary Fig. 6H, I).

The unweighted UniFrac distance matrix revealed clear clustering of microbial communities among different experimental groups, with both bacterial and fungal communities exhibiting substantial differences in species diversity between groups (Supplementary Fig.  7 A, C). Similarly, analysis based on the Bray–Curtis distance matrix showed distinct group-level clustering, with pairwise distances among samples within the same group (female ticks treated at −4 °C, male ticks treated at 27 °C) consistently low (light blue), indicating high intragroup community similarity (Supplementary Fig. 7B, D).

On the basis of the principal coordinate analysis (PCoA) of weighted UniFrac distances, the bacterial communities in male ticks treated at −4 °C and female ticks treated at −4 °C and 8 °C exhibited partial separation from those in the control groups (Supplementary Fig.  8 A). For the fungal communities, the PCoA of weighted UniFrac indicated a tendency of male ticks treated at −4 °C to separate from the control group (Supplementary Fig. 8B).

Analysis of similarities (ANOSIM) and nonparametric multivariate ANOVA (ADONIS) analyses further quantified these patterns. For bacteria, most pairwise comparisons yielded *R* values near zero or negative (Supplementary Table 13), and ADONIS indicated that temperature and sex explained only a small fraction of variance (*F* = 0.805, *R*^2^ = 0.266, *P* = 0.733; Supplementary Table 14), showing no significant effect. In contrast, fungal communities were significantly influenced by temperature (*F* = 2.611, *R*^2^ = 0.540, *P* = 0.001; Supplementary Table 14). These results indicate that cold stress has a limited impact on overall bacterial community structure but affects specific taxa, whereas fungal communities are more sensitive and undergo more pronounced compositional changes.

### Functional pathways of fungal metagenomes

**Fig 4.**
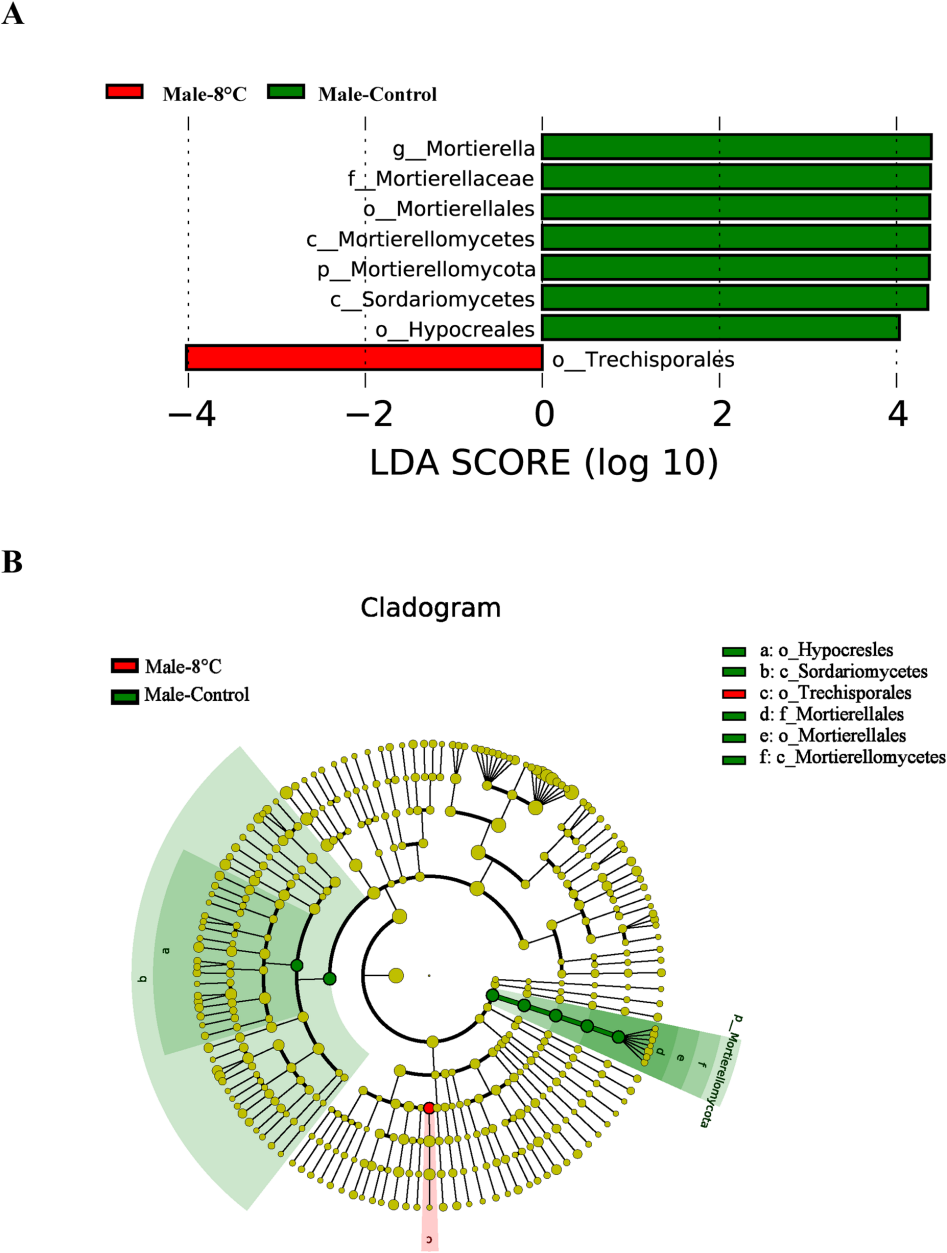
LEfSe analysis of *Dermacentor silvarum* microbiomes. **A** Histogram of the LDA scores computed for features differentially abundant among the male ticks treated at 8 °C and male ticks in control group for the fungal community. **B** A cladogram that denotes the taxa (highlighted by small circles and by shading) showing different abundance values (according to LEfSe) in two sampling groups

Linear discriminant analysis (LDA) effect size (LEfSe) yielded three major outputs (Fig. 4), including the effect sizes of differential features, the phylogenetic distribution of these differences, and the underlying raw data. LEfSe analyses were conducted across all temperature treatments in both female and male ticks. However, beyond the fungal biomarkers identified at 8 °C in males, no other bacterial or fungal taxa or predicted functional pathways met the predefined criteria for statistical significance (LDA > 4, *P* < 0.05) or exhibited biologically meaningful differentiation (Supplementary Table 15–17). To further examine functional differences, KEGG pathway profiles inferred from KO annotations were summarized at hierarchical levels (levels 1–3), and their relative abundances were expressed as percentages (Supplementary Table 18). Although no pathways showed statistically significant differences, several core functional categories—including transcription (RNA polymerase, transcription factors), translation (ribosome biogenesis), membrane transport (phosphotransferase system (PTS); ATP-binding cassette (ABC) transporters), DNA repair, stress response, and general metabolism—were consistently detected across groups. Notably, the relative abundance of membrane transport (PTS) and DNA repair pathways tended to be higher in certain low-temperature treatment groups (e.g., female ticks treated at −4 °C and male ticks treated at 0 °C) compared with controls, suggesting a temperature-associated response. Overall, these findings suggest that the predicted functional potential of the tick microbiome, particularly the bacterial component, remains relatively stable under the tested cold stress conditions. LEfSe analysis identified a total of seven fungal clades showing statistically significant and biologically consistent differences between the male ticks in the control group and male ticks treated at 8 °C (Fig. 4A, B).

In the male ticks in the control group, the most differentially abundant taxa belonged to the phylum Mortierellomycota, including Mortierellomycetes (class), Mortierellales (order), Mortierellaceae (family), and the genus *Mortierella* (Fig. 4A). These taxa displayed the highest positive LDA scores, indicating substantial enrichment under this condition. Conversely, the male ticks treated at 8 °C exhibited a marked enrichment of taxa within the order Trechisporales, which showed negative LDA scores (Fig. 4B). The cladogram further highlights the phylogenetic separation of these fungal lineages, with male ticks in the group-enriched taxa clustered predominantly within Mortierellomycota, while the enriched taxa in male ticks treated at 8 °C were concentrated within Trechisporales. These results indicate that low-temperature exposure induced distinct, treatment-specific shifts in fungal community composition, with Mortierellales prevailing in the male ticks in the control group and Trechisporales dominating the male ticks treated at 8 °C. Principal component analysis (PCA) ordinations of functional pathways showed a distinct cluster of female ticks treated at −4 °C and male ticks treated at −4 °C, which were clearly separated from other samples. (Supplementary Fig.  9 A). For the fungal functional pathways, PCA ordinations showed somewhat distinct clustering of male control ticks, female ticks treated at 8 °C, and male ticks treated at 8 °C, which were distanced from the other samples (Supplementary Fig. 9B).

## Discussion

Temperature is a key abiotic factor shaping the survival, distribution, and physiology of ectothermic arthropods, including ticks. Tick development and survival across life stages depend heavily on environmental temperature and humidity [32]. In this study, low-temperature exposure markedly altered both bacterial and fungal communities of *D. silvarum*, revealing temperature-driven microbial restructuring that may facilitate cold adaptation and overwintering survival. These findings align with previous studies showing that tick microbiota reflect interactions among environment, symbiont stability, and host physiology [12, 33].

Cold stress has been shown to selectively favor cold-tolerant and opportunistic microbes while suppressing thermosensitive taxa in a wide range of ectothermic organisms [9, 13]. In *I. ricinus*, seasonal environmental variations, including temperature fluctuations, significantly influence the composition and diversity of tick-associated microbial communities, with colder seasons often associated with higher richness and evenness of gut symbionts [13, 34]. Similar shifts in microbial diversity under environmental stress have also been observed in the microbiomes of insects. In the oriental fruit fly (*Bactrocera dorsalis*), the gut microbiota plays a crucial role in enhancing cold tolerance by regulating arginine and proline metabolism pathways [35]. Moreover, significant seasonal variations in the gut bacterial composition of insects have been reported; for instance, in field crickets, the abundance of *Pseudomonas* decreases while *Wolbachia* increases during overwintering [36]. In yellowfin tuna (*Thunnus albacares*), cold stress has been shown to increase microbial richness and alter gut microbial composition, indicating that cold exposure induces adaptive restructuring of the microbiota [37]. It is possible that the increased microbial diversity under cold conditions reflects a dynamic rebalancing toward taxa that support cold survival, such as the production of cryoprotectants or membrane lipid remodeling.

The beta diversity results further confirmed the profound impact of temperature on microbial community structure. A similar phenomenon has been observed in ticks, where seasonal and temperature-associated factors drive significant shifts in microbial community composition [12, 34], and experimental and functional studies have shown that specific symbionts can influence tick survival and development [38, 60].

Proteobacteria increased significantly under cold exposure, suggesting the proliferation of metabolically versatile taxa with high adaptive capacity. Notably, genera such as *Pseudomonas* and *Sphingomonas* are known to produce cryoprotective compounds, antifreeze proteins, and exopolysaccharides that stabilize cell membranes at low temperature [39, 40]. In other arthropods, *Pseudomonas* symbionts contribute to detoxification and stress resistance [41, 61]. The microbial community in *Dendroctonus ponderosae* is highly enriched in genes for terpene metabolism, especially within the genus *Pseudomonas*, which suggests a potential role for symbionts in overcoming host tree chemical defenses [61]. These parallels suggest that *Pseudomonas* in *D. silvarum* may play analogous detoxification or protective roles during cold exposure. Conversely, the relative abundance of *Coxiella*-like endosymbionts declined significantly under cold stress. These intracellular bacteria are essential for tick reproduction and nutrient metabolism, particularly in providing B vitamins [17, 42]. The downregulation of the obligate endosymbionts could potentially reduce reproductive efficiency or alter nutrient homeostasis. Nonetheless, this tradeoff might favor short-term survival under cold stress, reflecting an adaptive shift in host–symbiont dynamics.

*Ralstonia* detoxifies reactive nitrogen species through denitrification, and *Acinetobacter* possesses metabolic pathways for degradation of aromatic and aliphatic hydrocarbons [43–45]. These taxa may contribute to energy homeostasis and detoxification under cold stress [46, 47].

Genera such as *Aspergillus*, *Alternaria*, and *Candida* were prominent, suggesting that these taxa may be involved in the degradation or transformation of organic compounds under cold conditions. Many *Aspergillus* species possess enzymes capable of catalyzing the biotransformation of terpenes and other hydrocarbons [48]. Members of Ascomycota, such as *Grosmannia clavigera*, have demonstrated the capacity to detoxify monoterpenes and utilize them as carbon sources via β-oxidation and oxygenation pathways [49]. These fungi also upregulate ABC transporters (e.g., GcABC-G1) that mediate efflux of toxic monoterpenes [50], which may similarly operate within the cold-adapted tick mycobiome.

*Ganoderma* species are well recognized for producing lignocellulolytic and cell-wall-degrading enzymes that enable the breakdown of complex organic substrates derived from both plant and host sources during stress conditions [51]. In addition, yeasts of the genus *Trichosporon* exhibit the ability to metabolize monoterpenes, such as limonene, through hydroxylation and oxidation reactions, forming products, including isopiperitenone and (+)-limonene-1,2-trans-diol [52, 62]. The enrichment of *Trichosporon* in cold-treated samples suggests that these fungi may assist in maintaining cellular homeostasis through lipid modification and detoxification pathways. This interpretation aligns with previous findings showing that psychrotolerant yeasts employ adaptive mechanisms, such as membrane lipid remodeling, antioxidant enzyme activation, and cryoprotectant synthesis to preserve metabolic balance and structural integrity under low-temperature conditions [53, 54].

From a functional perspective, microbial shifts under cold stress are likely to trigger metabolic reprogramming that enhances host tolerance. Enriched taxa such as *Pseudomonas*, *Sphingomonas*, and *Trichosporon* may act together to support antioxidative defenses, biosynthesis of cryoprotectants, and membrane stabilization. For example, cold-adapted *Pseudomonas* strains produce exopolysaccharides (EPS) with demonstrable cryoprotective properties. EPS can lower water’s freezing point, reduce ice nucleation, and protect cells from desiccation and freeze damage. Empirical studies on cold-isolated *Pseudomonas* spp. and Antarctic strains have characterized EPS with clear cryoprotective activity and biotechnological potential [55, 56]. Similarly, psychrotolerant yeasts including taxa related to *Trichosporon* employ conserved cold-adaptive strategies, such as membrane lipid remodeling, upregulation of stress-responsive enzymes, and shifts in transcriptional programs that preserve membrane fluidity and redox balance under low temperatures [53, 54]. KEGG-based functional prediction indicated that pathways related to environmental responsiveness, stress tolerance, and DNA maintenance exhibited moderate variation under low-temperature conditions. In particular, increased proportions of membrane transport pathways (PTS and ABC transporters) imply enhanced substrate uptake and adaptation to environmental changes, while elevated DNA repair and stress-response functions may reflect increased demands for cellular stability under cold stress. In contrast, translation-related pathways (ribosome biogenesis) remained relatively stable, highlighting the preservation of core cellular processes. Overall, these findings suggest that the microbial communities adjust to low temperatures through coordinated modulation of transport, stress response, and metabolic pathways rather than broad shifts in transcription or translation functions. These microbial functions could synergize with host physiology to provide a cooperative mechanism for cold tolerance. Indeed, experimental work in* I. scapularis* demonstrates that bacterial (*Anaplasma phagocytophilum*) infection can modulate host gene expression, inducing an antifreeze glycoprotein and improving tick survival at low temperatures [38], and reviews of tick symbioses further highlight multiple ways endosymbionts influence tick fitness and stress responses [57]. Collectively, these findings support the hypothesis that the cold-induced enrichment of specific bacterial and fungal taxa contributes to the overwintering resilience of ticks through complementary detoxification, membrane stabilization, and cryoprotection mechanisms.

Although male and female *D. silvarum* were analyzed separately, the observed sex-specific microbiome responses to cold stress likely reflect differences in physiological demands and survival strategies. Females may rely more heavily on endosymbionts such as *Coxiella* to support nutrient provisioning for reproduction, whereas males may tolerate a broader spectrum of cold-tolerant or detoxification-associated microbes to prioritize immediate survival under low-temperature stress. These distinctions suggest that sex-specific microbial dynamics are biologically relevant, representing complementary strategies for overwintering resilience. Thus, the overall patterns of cold-induced microbial restructuring are consistent across sexes, whereas the subtle differences in taxa abundance may indicate differential allocation of microbial functions, including detoxification, cryoprotection, and membrane stabilization, according to sex-dependent physiological priorities.

Understanding these sex-specific interactions may provide insights into vector competence, reproductive fitness, and ecological adaptation of ticks under seasonal environmental stress. Moreover, manipulation of tick microbiota has been proposed as a potential strategy for controlling ticks and tick-borne pathogens, as targeting keystone gut bacteria with anti-microbiota vaccines can alter microbial community structure and reduce pathogen colonization [58]. Immunization against key microbial taxa such as Enterobacteriaceae has also been shown to affect tick physiology and microbiota assembly, suggesting a promising avenue for integrated tick management [59]. Given that all ticks were handled identically and randomly assigned to temperature treatments, and all samples were processed simultaneously using consistent extraction and amplification protocols, the initial microbiome composition may have differed across groups. Therefore, the observed differences in ASV counts, particularly among low-abundance taxa, likely reflect the high resolution and sensitivity of ASV-based inference, stochastic fluctuations during incubation, and sequencing depth, rather than temperature-induced colonization of novel taxa. It is important to include sequencing data from negative controls for DNA extraction or the incubation environment, which represents a limitation of the current research.

In summary, cold stress drives comprehensive reorganization of *D. silvarum* microbiota, characterized by a reduction in nutrient-provisioning *Coxiella* symbionts, enrichment of psychrotolerant taxa including *Pseudomonas* and *Sphingomonas*, and expansion of detoxification-associated fungi including *Aspergillus* and *Trichosporon*. This restructured microbial community may enhance tick resilience to cold, oxidative, and osmotic stress, contributing to overwintering success. Understanding the specific metabolic interactions between *D. silvarum* and its microbiota under cold stress will provide valuable insights into tick ecology, vector competence, and potential targets for biological control.

## Conclusions

This study demonstrates that low-temperature exposure drives pronounced restructuring of the bacterial and fungal microbiota in *D. silvarum*. Cold stress was associated with a reduction in nutrient-provisioning symbionts and an enrichment of cold-tolerant and metabolically flexible taxa, suggesting functional shifts toward cryoprotection, membrane stabilization, and detoxification. These findings highlight the microbiome as an integral component of tick cold adaptation and overwintering survival. Overall, our results provide new insights into the ecological significance of temperature-driven microbiome dynamics and establish a basis for future studies on microbe-mediated mechanisms underlying tick fitness and vector competence under environmental stress.

## Supplementary Information

Additional file 1.

Additional file 2.

Additional file 3.

Additional file 4.

Additional file 5.

Additional file 6.

Additional file 7.

Additional file 8.

Additional file 9.

Additional file 10.

Additional file 11.

Additional file 12.

Additional file 13.

Additional file 14.

Additional file 15.

Additional file 16.

Additional file 17.

Additional file 18.

Additional file 19: Supplementary Figure 1. Species accumulation curves for the bacterial communityand fungal community.

Additional file 20: Supplementary Figure 2. Rarefaction curves of Shannon’s index for the bacterial communityand fungal community. DSFC: female control group, and three replicates, FC1, FC2, and FC3; DSF8: female ticks treated at 8 °C for 7 days, and three replicates, F81, F82, and F83; DSF4: female ticks treated at 4 °C for 7 days, and three replicates, F41, F42, and F43; DSF0: female ticks treated at 0 °C for 7 days, and three replicates, F01, F02, and F03; DSF04: female ticks treated at −4 °C for 7 days, and three replicates, F041, F042, and F043; DSMC: male control group, and three replicates, MC1, MC2, and MC3; DSM8: male ticks treated at 8 °C for 7 days, and three replicates, M81, M82, and M83; DSM4: male ticks treated at 4 °C for 7 days, and three replicates, M41, M42, and M43; DSM0: male ticks treated at 0 °C for 7 days, and three replicates, M01, M02, and M03; DSM04: male ticks treated at −4 °C for 7 days, and three replicates, M041, M042, and M043.

Additional file 21:Supplementary Figure 3. Venn diagrams of the microbiota in *Dermacentor silvarum*, showing the number of shared and unique amplicon sequence variantsamong samples subjected to different low-temperature treatments and control groups.Bacterial ASVs;fungal ASVs.

Additional file 22: Supplementary Figure 4. Relative abundance of the top ten dominant bacterialand fungalspecies in *Dermacentor silvarum* samples under different low-temperature treatments.

Additional file 23: Supplementary Figure 5. Differential bacterial composition among low-temperature treatment groups. Significant differences between groups were assessed using Student’s *t*-test, with *P* < 0.05 considered statistically significant, as indicated by the color scale. Pairwise comparisons were performed at the genus level as follows:females control versus females treated at 8 °C;males control versus males treated at 4 °C;males control versus males treated at 0 °C;females control versus females treated at 4 °C for the fungal community;females control versus females treated at −4 °C;females control versus females treated at 8 °C;males control versus males treated at 0 °C;males control versus males treated at −4 °C; andmales control versus males treated at 8 °C.

Additional file 24: Supplementary Figure 6. Hierarchical cluster heatmap showing the most abundant familiesof bacteriaand fungi.

Additional file 25: Supplementary Figure 7. Beta diversity index Bray_curtis and Unweighted unifracdistance matrix heatmap of the bacterial diversityand fungal diversity. DSFC: female control group, and three replicates, FC1, FC2, and FC3; DSF8: female ticks treated at 8 °C for 7 days, and three replicates, F81, F82, and F83; DSF4: female ticks treated at 4 °C for 7 days, and three replicates, F41, F42, and F43; DSF0: female ticks treated at 0 °C for 7 days, and three replicates, F01, F02, and F03; DSF04: female ticks treated at −4 °C for 7 days, and three replicates, F041, F042, and F043; DSMC: male control group, and three replicates, MC1, MC2, and MC3; DSM8: male ticks treated at 8 °C for 7 days, and three replicates, M81, M82, and M83; DSM4: male ticks treated at 4 °C for 7 days, and three replicates, M41, M42, and M43; DSM0: male ticks treated at 0 °C for 7 days, and three replicates, M01, M02, and M03; DSM04: male ticks treated at −4 °C for 7 days, and three replicates, M041, M042, and M043.

Additional file 26: Supplementary Figure 8. Principal coordinate analysisof weighted UniFrac of the bacterial diversityand fungal diversity. DSFC: female control group, and three replicates, FC1, FC2, and FC3; DSF8: female ticks treated at 8 °C for 7 days, and three replicates, F81, F82, and F83; DSF4: female ticks treated at 4 °C for 7 days, and three replicates, F41, F42, and F43; DSF0: female ticks treated at 0 °C for 7 days, and three replicates, F01, F02, and F03; DSF04: female ticks treated at −4 °C for 7 days, and three replicates, F041, F042, and F043; DSMC: male control group, and three replicates, MC1, MC2, and MC3; DSM8: male ticks treated at 8 °C for 7 days, and three replicates, M81, M82, and M83; DSM4: male ticks treated at 4 °C for 7 days, and three replicates, M41, M42, and M43; DSM0: male ticks treated at 0 °C for 7 days, and three replicates, M01, M02, and M03; DSM04: male ticks treated at −4 °C for 7 days, and three replicates, M041, M042, and M043.

Additional file 27: Supplementary Figure 9. Principal component analysisordinations of functional pathways for the bacterial communityand fungal community. DSFC: female control group, and three replicates, FC1, FC2, and FC3; DSF8: female ticks treated at 8 °C for 7 days, and three replicates, F81, F82, and F83; DSF4: female ticks treated at 4 °C for 7 days, and three replicates, F41, F42, and F43; DSF0: female ticks treated at 0 °C for 7 days, and three replicates, F01, F02, and F03; DSF04: female ticks treated at −4 °C for 7 days, and three replicates, F041, F042, and F043; DSMC: male control group, and three replicates, MC1, MC2, and MC3; DSM8: male ticks treated at 8 °C for 7 days, and three replicates, M81, M82, and M83; DSM4: male ticks treated at 4 °C for 7 days, and three replicates, M41, M42, and M43; DSM0: male ticks treated at 0 °C for 7 days, and three replicates, M01, M02, and M03; DSM04: male ticks treated at −4 °C for 7 days, and three replicates, M041, M042, and M043.

## Data Availability

The raw data were deposited to the Sequence Read Archive (SRA) of the NCBI (BioProject ID: PRJNA1069662, PRJNA1092565). Data supporting the main conclusions of this study are included in the manuscript.
